# The Theory of Fowler's Posture

**Published:** 1914-07-18

**Authors:** 


### The Theory of Fowler's Posture.

The propped-up position in the after-treatment of patients who have been operated on for any condition of acute peritonitis is firmly established in favour with surgeons. But according to Dandy and Rowntree, who have published some researches on the absorption of fluids from the peritoneum in the *Annals of Surgery*, the theoretical advantages which Dr. Fowler sought to secure by his posture rested upon two assumptions which have been proved to be quite false. These assumptions are that absorption from the peritoneum takes place into the lymphatic system, and that it is most active on the surface of the diaphragm. Dandy and Rowntree demonstrate that absorption is almost entirely into the blood stream, not into the lymphatics: the once famous stomata on the surface of the peritoneum are discredited as artefacts. Also that the visceral peritoneum absorbs fluids freely, as well as the mural layer. Absorption is equal in all postures except pelvis down, in which it is 15 per cent. less than in others. Although, therefore, Fowler's posture was based upon entirely fallacious views and reasoning, it does all the same result in slightly less active peritoneal absorption, and may therefore be useful in practice for this reason.

